# Assessment of Protein Nutritional Quality of Novel Hairless Canary Seed in Comparison to Wheat and Oat Using In Vitro Static Digestion Models

**DOI:** 10.3390/nu15061347

**Published:** 2023-03-10

**Authors:** Lamia L’Hocine, Allaoua Achouri, Emily Mason, Mélanie Pitre, Delphine Martineau-Côté, Stéphane Sirois, Salwa Karboune

**Affiliations:** 1 Agriculture and Agri-Food Canada, Saint-Hyacinthe Research and Development Centre, Saint-Hyacinthe, QC J2S 8E3, Canada; 2 Department of Food Science and Agricultural Chemistry, Macdonald Campus, McGill University, 21111 Lakeshore, Ste-Anne-de-Bellevue, QC H9X 3V9, Canada

**Keywords:** hairless canary seed, anti-nutritional factors, nutritional quality, amino acid, digestibility, pH-drop, INFOGEST, in vitro human digestion model, PDCAAS, DIAAS

## Abstract

Hairless canary seed (*Phalaris canariensis* L.) is a novel true cereal that is now approved for human consumption in Canada and the United States. This true cereal grain has higher protein content (22%) than oat (13%) and wheat (16%) and represents a valuable source of plant proteins. Assessment of canary seed protein quality is therefore essential to evaluate its digestibility and ability to provide sufficient amounts of essential amino acids for human requirements. In this study, the protein nutritional quality of four hairless canary seed varieties (two brown and two yellow) were evaluated in comparison to oat and wheat. The assessment of anti-nutrients contents (phytate, trypsin inhibitor activity, and polyphenols) showed that brown canary seed varieties had the highest content in phytate and oat the highest in polyphenols. Trypsin inhibitor level was comparable among studied cereals, but slightly higher in the brown canary seed Calvi variety. In regard to protein quality, canary seed had a well-balanced amino acid profile and was particularly high in tryptophan, an essential amino acid normally lacking in cereals. The in vitro protein digestibility of canary seeds as determined by both the pH-drop and INFOGEST (international network of excellence on the fate of food in the gastrointestinal tract) protocols appears slightly lower than wheat and higher than oat. The yellow canary seed varieties showed better overall digestibility than the brown ones. For all studied cereal flours, the limiting amino acid was lysine. The calculated in vitro PDCAAS (protein digestibility corrected amino acid score) and DIAAS (digestible indispensable amino acid score) were higher for the yellow C05041 cultivar than the brown Bastia, similar to those of wheat, but lower than those of oat proteins. This study demonstrates the feasibility and utility of in vitro human digestion models for the assessment of protein quality for comparison purpose.

## 1. Introduction

De-hulled hairless (glabrous) canary seeds (*Phalaris canariensis* L.) were given GRAS status and approved for human consumption in Canada and the United States as a true cereal grain in 2016 [1,2]. In comparison to other prevalent cereal grains, such as wheat, oat, barley, and rye, canary seed has a higher protein content of 22% with a reported higher amount of the essential amino acid tryptophan, which is normally deficient in cereals [3]. This makes hairless canary seed an interesting source of plant proteins. From a dietary standpoint, assessment of canary seed protein quality is essential to evaluate its ability to provide sufficient amounts of essential amino acids for human requirements. Protein quality is not only defined by its composition in essential amino acids, but also by its digestibility [4]. Digestibility is often used to estimate amino acid bioavailability [5]. The digestibility of plant proteins can in turn be influenced by the presence of anti-nutritional factors, the composition of the food matrix and by processing [6]. There are several scores and methods that have been developed and implemented for the assessment of protein quality. The protein digestibility corrected amino acid score (PDCAAS) and the digestible indispensable amino acid score (DIAAS) values are measurements of protein quality and provide information regarding the ability of a specific protein to provide sufficient amounts of essential amino acids for human requirements [7]. The PDCAAS method has been the recommended measure of protein quality by the Food and Agriculture Organization of the United Nations (FAO) since 1989 [4]. However, since 2013, the DIAAS is established as the successor to PDCAAS [8] due to several concerns. In particular, the PDCAAS is based on an estimate of crude protein digestibility determined over the total digestive tract and assumes that all amino acids (AA) have the same digestibility as the total protein. The DIAAS, instead, considers dietary amino acids as individual nutrients, and therefore data need to be given on the individual dietary amino acid digestibility at the end of the small intestine (the ileum). It is now recognized that the PDCAAS generally underestimates the value of high-quality proteins and overestimates the value of low-quality proteins [9,10]. The PDCAAS is typically based on a rodent model; the pig has been recognized, however, as a more appropriate model than rats for estimating protein and AA digestibility in foods for humans [9,11,12]. Animal in vivo studies are complex, time consuming, costly, and pose ethical concerns. As well, they may not accurately reflect the digestibility of amino acids that occurs in humans. Instead, in vitro assays are considered less expensive to conduct, not subjected to ethical constraints, and can reduce variability related to biological differences among subjects [13,14]. In this context, and in concordance with the principles of the Three Rs—Replacement, Reduction, and Refinement—in animal experiments [15], advancing standardized human in vitro digestibility methods for assessing protein digestibility are sought to potentially enable the calculation of PDCAAS and DIAAS for conventional and novel dietary proteins without the need for animal bioassays. Therefore, the overall objective of this study was to first determine the amino acid composition and anti-nutritional components of the newly developed Canadian hairless canary seed varieties, and to evaluate their protein nutritional quality as compared to proteins from selected common cereal grains oat (*Avena sativa*) and wheat (*Triticum estivum* L.) by two different human in vitro static digestion models. The simple pH-drop protocol of Tinus et al. [16], and the more complex harmonized INFOGEST (international network of excellence on the fate of food in the gastrointestinal tract) protocol [17] were used to determine total protein and amino acid ileal digestibility of canary seed, oat, and wheat flours and subsequently calculate their respective protein quality scores PDCAAS and DIAAS. There are only a few recent studies that have applied the INFOGEST protocol to the assessment of nutritional quality of dietary proteins [18,19]. To the best of our knowledge, this is the first study that uses the INFOGEST protocol to determine AA and protein quality scores (DIAAS) of the novel canary seed, wheat, and oat flours.

## 2. Materials and Methods

### 2.1. Materials

Four hairless dehulled canary seed (*Phalaris canariensis* L.) cultivars were used in this study. These varieties were developed and produced by the Crop Development Center (CDC) of the University of Saskatchewan (Saskatoon, SK, Canada) [20], namely the yellow cultivars C09052 and C05041 (now registered as cultivar CDC Cibo), and the brown CDC Calvi and CDC Bastia cultivars. Oat (*Avena sativa*) cultivar Turcotte and wheat (*Triticum estivum* L.) cultivar Snowbird were purchased from Semican (Princeville, QC, Canada) and used for comparison purpose in this study. All seeds were hand-cleaned to remove any broken seeds or foreign material. 

HPLC grade organic solvents, trinitrobenzenesulfonic acid (TNBS), internal standard norvaline, and Halt protease inhibitor were purchased from Fisher Scientific (Fair Lawn, NJ, USA). Buffers, L-leucine, sodium bicarbonate (NaHCO_3_), monosodium phosphate (NaH_2_PO_4_), 4-(2-aminoethyl) benzenesulfonyl fluoride hydrochloride (AEBSF), and disodium phosphate (Na_2_HPO_4_) were purchased from BioShop (Burlington, ON, Canada). Folin–Ciocalteu’s phenol reagent, Nα-benzoyl-DL-arginine 4-nitroanilide hydrochloride (BAPNA), ferulic acid, phenol, calcium chloride (CaCl_2_), ammonium acetate (C_2_H_7_NO_2_), borax (Na_2_B_4_O_7_·10H_2_O), sodium acetate (C_2_H_3_NaO_2_), and sodium azide (NaN_3_) were purchased from Sigma (St. Louis, MO, USA). 

For the digestion work, pancreatin from porcine mucosa (P7545), pepsin from porcine gastric mucosa (250 units/mg solid, P7000), α-amylase from porcine pancreas (10 units/mg solid, A3176), and trypsin (T0303) were purchased from Sigma (St. Louis, MO, USA). Deionized water (Millipore, Burlington, MA, USA) was used in all experiments.

### 2.2. Preparation and Determination of Protein Content of Canary Seed, Oat and Wheat Flours

Canary seeds, oat, and wheat flours were prepared by grinding dehulled seeds into fine powders in liquid nitrogen using an analytical mill (IKA A11, IKA, Staufen, Germany). The flours were stored at 4 °C in the dark until used for analysis. Total nitrogen content of canary seed, oat, and wheat flours were determined using a Vario MAX Cube (Elementar, Langenselbold, Germany), following the Dumas combustion method [21] with EDTA as a nitrogen standard. Crude protein content of cereal flours was calculated as total nitrogen multiplied by a conversion factor of 5.7 for canary seed and wheat [1], and 6.25 for oat.

### 2.3. Determination of Anti-Nutritional Components of Canary Seed, Oat, and Wheat Flours

#### 2.3.1. Trypsin Inhibitor Activity (TIA)

TIA was assessed according to Makkar et al. [22], with modification. Cereal flours were defatted with ethanol. The dried, defatted flours were ground into a fine powder and stored at −20 °C until analysis. Then, 50 mL of 0.01 M NaOH was added to 4 g of defatted flour and stirred for 3 h at room temperature. The pH of the solution was adjusted to 9.5 using concentrated NaOH or HCl. A 15 mg/L trypsin solution was prepared daily in 0.001 N HCl. The BAPNA substrate was prepared daily by dissolving 40 mg in 1 mL of DMSO and then diluted to 100 mL (0.921 mM) with pre-warmed (37 °C) 0.05 M Tris-HCl buffer, pH 8.2, containing 0.02 M CaCl_2_. A reagent blank, standard enzyme solution, sample blank, and sample solution were prepared for the assay. The reagent blank (a) contained 2 mL of distilled water. The standard (b) contained 2 mL of the standard trypsin solution (15 mg/L trypsin) and 2 mL of distilled water. The sample blanks (c) contained 1 mL of diluted sample extract plus 1 mL of distilled water. The samples (d) contained 1 mL of diluted sample extract, 1 mL of distilled water, and 2 mL of trypsin solution. 

The tubes were vortexed and preheated in a water bath at 37 °C for 10 min. Then, 5.0 mL of BAPNA solution (pre-incubated at 37 °C) was added to each tube. After 10 min of incubation at 37 °C, 1.0 mL of 30% (*v*/*v*) acetic acid was added to each tube to stop the reaction and 2.0 mL of trypsin solution was added to the reagent blank (a) and sample blank (c) tubes. All tubes were centrifuged at 3000× *g* for 10 min at room temperature and the absorbance of each solution was read at 410 nm. The change in absorbance (Ai) due to trypsin inhibitor/mL diluted sample extract was calculated from the following equation:

Ai = (Ab − Aa) − (Ad − Ac)

where Ab, Aa, Ad, and Ac are the absorbance readings of the standard, reagent blank, samples, and sample blanks, respectively. The percent inhibition of each sample tube was calculated from the following equation:

% Trypsin inhibition = Ai/(Ab − Aa)

Since 1 μg of pure trypsin gives an absorbance of 0.019, trypsin inhibitor activity (TIA) was expressed in terms of mg of pure trypsin inhibited per gram of sample (mg/g) and was calculated from the following equation:

TIA (mg/g) = (2.362 × A_i_ × DF)/S

where DF is the dilution factor and S is the sample weight in grams. 

#### 2.3.2. Phytate Content

Determination of phytate content was carried out according to McKie and McCleary [23] using a commercial assay kit (Megazyme International, Bray, Wicklow, Ireland). Briefly, 20 mL of HCl (0.66 M) was added to 1 g of cereal flours and stirred overnight at room temperature for acid extraction of inositol phosphates. The extracted inositol phosphates were subsequently treated with phytase and phosphatase enzymes to convert total phosphate to inorganic phosphorous. The amount of inorganic phosphorus released was determined from its reaction with ammonium molybdate, which forms molybdate blue proportional to the amount of inorganic phosphorous present in the sample. Molybdate blue content was determined colorimetrically at 655 nm from a standard curve using known concentrations of inorganic phosphorus. The assay determines the g of phosphorous in 100 g of sample material, based on the assumption that the amount of phosphorous measured is exclusively released from phytate. The results were expressed as mg of phytate per gram of sample. 

#### 2.3.3. Total Polyphenol Content (TPC)

TPC was determined using the Folin–Ciocalteu reagent according to Singleton and Rossi [24], with modification. Samples (flours) (5%, *w*/*v*) were extracted for 2 h at room temperature with 70% ethanol containing 1% (*v*/*v*) concentrated HCl. The mixtures were centrifuged at 10,000× *g* for 15 min and the supernatants were recovered. Ferulic acid (50–500 mg/L), prepared in 70% ethanol containing 1% (*v*/*v*) concentrated HCl, was used to construct the standard curve. Then, 1.5 mL of Folin–Ciocalteu reagent (diluted 10× with water) was added to 200 μL of blanks, standards, and samples followed by the addition of 1.5 mL sodium bicarbonate solution 7.5% (*w*/*v*) after 5 min (at room temperature). After an additional 90 min at room temperature, the sample tubes were centrifuged at 6000× *g* for 15 min, and the absorbance of the supernatants were read at 750 nm. The TPC content in the samples was determined from the ferulic acid standard curve, and results were expressed as mg ferulic acid equivalents (FAE)/g of flour.

### 2.4. Determination of Amino Acid Content of Canary Seed Flours and In Vitro Digestates 

Total amino acid analysis of canary seed flours and freeze-dried supernatant hydrolysates was conducted in accordance with the Agilent method [25]. Briefly, samples containing 4 mg of protein (30 mg flour) were hydrolyzed with 6 N HCl containing 0.1% (*w*/*v*) phenol and Norvaline (as internal standard) for 24 h at 110 ± 2 °C in glass tubes sealed under vacuum. The hydrolyzed samples were cooled to room temperature, and solutions evaporated with nitrogen to dryness. Once dry, the amino acids were dissolved by the addition of 10 mM borax buffer (pH 8.2, containing 0.1% *w*/*v* HCl) and then filtered with 0.22 µm PVDF filters (low protein binding) (Sigma, St. Louis, MO, USA) prior to RP-HPLC analysis. For the in vitro digestates, 200 μL of the supernatant was removed and analyzed for free soluble (bioaccessible) amino acids. The remaining supernatant was lyophilized and subsequently hydrolyzed following the same protocol as canary seed flour for the determination of total amino acids in the whole in vitro digestate, which includes free amino acids as well as polypeptides and soluble proteins. 

Amino acid composition was quantified by RP-HPLC analysis using an Agilent Poroshell HPH-C18 reversed-phase column (monitored with Agilent 1200 series HPLC system (Agilent Technologies Inc., Mississauga, ON, Canada), utilizing an automatic post-column OPA and FMOC derivatization and detection at an absorbance of 338 nm. The separation was performed at a flow rate of 1.5 mL/min employing a mobile phase of A: 10 mM Na_2_HPO_4_, 10 mM Na_3_B_4_O_7_, 5 mM NaN_3_, adjusted to pH 8.2 with HCl, and B: ACN: MeOH: water (45:45:10, *v*/*v*/*v*). The elution program was as follows: 0 min, 2% B; 1.0 min, 2% B; 20 min, 59% B; 21 min, 90% B; 24 min 90% B; 29 min, 2% B; 35 min, 2% B. Five standard mixture ampoules (containing 16 amino acids) at different concentrations (10 pmoles/μL to 1 nmoles/μL) from Agilent with the addition of norvaline as an internal standard (IS) were used for the construction of the calibration curves by using the ratio of responses for the standard and analytes. The elution times of each amino acid in the analyzed samples were compared to those of the standard, and the concentration of each amino acid (pmoles/μL) was calculated based on the peak area. Amounts were then expressed in mg amino acid/100g protein based on each amino acid molecular weight and protein content of flour sample. 

The content of tryptophan in the canary seed flours was determined separately by alkali hydrolysis following the method of Yust et al. [26] with slight modification. Samples (~15 mg of protein) were dissolved in 3 mL of 4 N NaOH, sealed in hydrolysis tubes, and incubated in an oven at 110 °C for 24 h. Hydrolysates were cooled, neutralized to pH 7.0 using 12 N HCl, and diluted to 25 mL with 1 M borax buffer (pH 9). Aliquots of these solutions were filtered through a 0.45 µm PVDF filter, and then injected into a Nova-Pack C18 column (Waters, Mississauga, ON, Canada). An isocratic elution system consisting of 25 mM sodium acetate and 0.02% sodium azide (pH 9)/acetonitrile (91:9, *v*/*v*) delivered at 1 mL/min was used. Tryptophan standard was injected at different concentrations for calibration construction, and the amount of tryptophan in flour samples was then calculated as mg/g based on the peak area. 

### 2.5. Determination of Total In Vitro Protein Digestibility by the pH-Drop In Vitro Digestion Model 

For the determination of the in vitro PDCAAS of the cereal flours, total protein digestibility (IVPD) was estimated according to the method described by Tinus et al. [16]. Briefly, cereal flour equivalent to 62.5 mg of protein was rehydrated in 10 mL of water at 37 °C for 1 h; afterwards, the pH of the solution was adjusted to 8.0 with 0.1 M NaOH and/or HCl. A 10 mL multienzyme solution was prepared fresh daily, consisting of 16 mg of trypsin (T0303 trypsin from porcine pancreas, type IX-S, 13,000–20,000 BAEE units/mg protein), 31 mg of chymotrypsin (C4129 Chymotrypsin from bovine pancreas C4129 Type II, ≥40 units/mg protein) and 13 mg protease (P5147 protease from Streptomyces griseus, Type XIV, ≥3.5 units/mg solids). The multienzyme solution was kept at 37 °C, and its pH was adjusted to 8.0 with 0.1 M NaOH and/or HCl. After rehydration, 1 mL of the multi-enzyme solution was added to the 10 mL sample mixture, and the initial pH was immediately recorded. After 10 min of constant agitation at 37 °C, the final pH was recorded and the IVPD was calculated from the following equation:

IVPD (%) = 65.66 + 18.10 ∆pH10 min

### 2.6. Determination of In Vitro Ileal Protein and Amino Acid Digestibility of Canary Seed, Oat, and Wheat Flours by the Harmonized INFOGEST Digestion Model 

The cereal flours were digested according to the method of INFOGEST consensus protocol as described by Minekus et al. [17]. In the oral phase of digestion, 1 g of cereal flour (canary seed, oat, and wheat) was incubated for 2 min at 37 °C, pH 7.0, with simulated salivary fluid (SSF) (1:1) containing α-amylase from porcine pancreas (75 U/mL of digestate). Then, the mixture was diluted (1:1, *v*/*v*) with simulated gastric fluid (SGF) containing pepsin from porcine gastric mucosa (2000 U/mL digestate). The pH of the mixture was adjusted to pH 3.0 and then incubated for 2 h at 37 °C. The intestinal phase was carried out by diluting the mixture (1:1, *v*/*v*) with simulated intestinal fluid (SIF) containing pancreatin from porcine mucosa (100 U trypsin activity/mL digestate) and bile (10 mM). The pH of the mixture was adjusted to pH 7.0 and incubated for 2 h at 37 °C. The reaction was stopped by placing the solutions on ice and adding 1-mM AEBSF (protease inhibitor). The final digestates were centrifuged (15,000× *g*, 30 min, 4 °C); the supernatants were collected and then frozen at −80 °C until analysis. 

### 2.7. Determination of Protein Degree of Hydrolysis (DH) 

The extent to which cereal flour proteins were hydrolyzed following the in vitro digestion protocol of Minekus et al. [17] was quantified using the TNBS reagent according to the method described by Adler-Nissen [27] and Spellman et al. [28], with modification. Briefly, 10 µL of sample and standard (both prepared in 0.1% (*w*/*v*) SDS) was added to a microplate well followed by 80 µL of 0.2125 M sodium phosphate buffer, pH 8.2, and 80 µL of 0.1% TNBS reagent, diluted with water. After mixing, the samples were incubated at 50 °C for 60 min. Then, 160 µL of 0.1 N HCl was added to stop the reaction and the absorbance read at 340 nm. Next, 0–2 mM of L-Leucine, prepared and diluted in 1% SDS, was used to generate the standard curve. The DH values were calculated using the following equation:

DH (%) = 100 × (AN_2_ − AN_1_)/N_pb_

where AN_1_ is the amino nitrogen content of the protein substrate before hydrolysis (mg/g protein), AN_2_ is the amino nitrogen content of the protein substrate after hydrolysis (mg/g protein), and N_pb_ is the nitrogen content of the peptide bonds (mg/g protein) after complete hydrolysis with 6 N HCl at 110 °C for 24 h. The values of AN_2_, AN_1_, and N_pb_ were determined from the standard curve of the absorbance at 340 nm versus the mg/L amino nitrogen content of L-leucine. The values obtained were then divided by the protein content in the test samples to give mg amino nitrogen per g of protein.

### 2.8. Calculations of the In Vitro PDCAAS and DIAAS for Protein Quality Evaluation 

The PDCAAS and DIAAS scores were calculated using the total protein and amino acid digestibility. The total protein digestibility was determined using both the pH-drop protocol (IVPD-T) [16] and the INFOGEST protocol (IVPD-M) [17], while the in vitro true ileal amino acid digestibility (IV-TID) was determined only by the INFOGEST method. The scores were calculated following the new FAO guidelines for determination of dietary protein quality for infants (0–6 months), children (6 months–3 years), and older children/adolescents/adults according to the recommended reference scoring patterns [8], since essential amino acid requirements for maintenance and growth are different for each age groups (Table 1). 

The amino acid content and the in vitro protein digestibility (IVPD) were used to calculate the PDCAAS of each cereal flour as by the following equation:

PDCAAS (%) = 100 × lowest value [(mg of indispensable amino acid in 1 g of the dietary protein)/(mg of the same dietary indispensable amino acid in 1 g of the reference protein)] × in vitro protein digestibility (IVPD)

For the DIAAS, both free and total amino acid content in the INFOGEST digestates of the cereal flours were quantified to determine what is designated as the minimum and maximum values for the DIAAS, respectively. It was hypothesized that the measurement of the free amino acids content in the digestate supernatant represents the amount of AA that are readily accessible for absorption and would therefore estimate the minimum DIAAS value. Meanwhile, the maximum DIAAS value was estimated by quantifying total amino acids after acidic hydrolysis of the digestate’s supernatant (including both bioaccessible free AA and soluble proteins/polypeptides) and which would correspond to the maximum true ileal digestibility (TID) of each amino acid. It is noted here that free and total AA brought by the digestion model components, i.e., digestive enzymes and solutions were quantified through blank control runs and subtracted as reflected in the equation by Havenaar et al. [29] below, which was used to calculate the in vitro true ileal amino acid digestibility (IV-TID).

In vitro true ileal AA digestibility (%) = ∑AA content sample digestate (mg) − ∑AA content blank (mg)/Intake AA content (mg)

where ∑AA content sample digestate is the total amino acid content (mg) in the supernatant after in vitro digestion; ∑AA content in sample blank is the amino acid content (mg) of the blank supernatant (containing all enzymes and solutions of the in vitro digestion without the addition of sample) after in vitro digestion; and Intake AA content is the amino acid content (mg) of the starting material (flour).

The digestible dietary indispensable amino acid content for both free and total amino acids was used to calculate the DIAAS for each cereal flour using the following equation:

DIAAS (%) = 100 × lowest value [(mg of digestible dietary indispensable amino acid in 1 g of the dietary protein)/(mg of the same dietary indispensable amino acid in 1 g of the reference protein)]

It is expected that the real DIAAS value lies between the measured minimum (free amino acids) and maximum (total amino acids) values. 

### 2.9. Statistical Analysis 

Each experiment was run in triplicate, and the data were expressed as means ± standard deviation. Statistical analyses were performed using XLSTAT software (Addinsoft, New York, NY, USA) in Microsoft Excel (Redmond, WA, USA). One-way analysis of variance (ANOVA) and the Tukey’s honest significant difference (HSD) test (*p* < 0.05) were performed to detect significant differences. For the AA digestibility and DIAAS values, statistical analysis was performed using SAS software (Cary, NC, USA). ANOVA was determined using the MIXED procedure of the SAS system. Multiple comparisons were performed with the LSMEANS statement of the MIXED procedure using the Bonferroni option.

## 3. Results and Discussion

### 3.1. Characterization of Dehulled Canary Seed, Oat and Wheat Flours 

Dehulled canary seed flour protein content ranged from 21.9–22.5% (*w*/*w*), with no significant difference between the two yellow and brown cultivars (Table 2). However, the protein content of the canary seed flours was significantly higher (*p* < 0.05) than those of oat (14.3%, *w*/*w*) and wheat (16.35%, *w*/*w*) flours, thereby exceeding most commonly consumed cereal grains, for which protein content generally ranges between 10% to 15% of the dry grain [30]. 

Cereals possess several anti-nutritional components that can reduce the digestion, bioavailability, and absorption of proteins, peptides, and amino acids, and hence affect their overall quality. The most common being trypsin inhibitor, phytate, and tannins [31]. In these regards, trypsin inhibitor activity (TIA), the phytate, and the total polyphenol content (TPC) were quantified in the investigated cereals. Trypsin inhibitor activity expressed in canary seed flours was comparable to that present in oat and wheat (Table 2). Wheat had a significantly lower TIA (0.114 mg/g) than the brown canary seed cultivar Calvi (0.161 mg/g) but not significantly different (*p* > 0.05) from the other canary seed cultivars and oat. Similarly, Abdel-Aal, et al. [32] reported no significant difference between the trypsin inhibitor content in a hairless brown variety (CDC Maria) as compared to wheat. On the other side, phytate content (Table 2) in canary seed flours was the highest, followed by oat flour, and then wheat. There was, however, no significant difference (*p* > 0.05) in phytate content between the studied canary seed varieties. Abdel-Aal et al. [32] also reported a higher content of phytate in a hairless brown cultivar of canary seeds (18.8 mg/g) as compared to wheat (10.7 mg/g). However, the phytate content in canary seed flour is comparable to that reported for other cereals such as buckwheat (9.2–16.2 mg/g), amaranth (10.6–15.1 mg/g), sorghum (5.9–11.8 mg/g), and legumes such as black beans (8.5–17.3 mg/g), kidney beans (8.3–13.4 mg/g), and soybeans (9.2–16.7 mg/g) [33]. The phytate content of cereals is influenced by cultivar, climatic conditions, and year. In addition, most phytate is located in the outer parts of the kernel, and different products of milling contain different levels of phytate depending on the raw material (dehulled or non dehulled) and method of processing [34]. Phytate, although crucial for plants, is considered as an antinutrient because it reacts with proteins [35], starch [36], and divalent cations such as Zn, Fe, and Ca to form insoluble complexes which reduce their functionality, bioavailability, nutritional value, and absorption [37].

For the total polyphenol contents (TPC), no significant differences were observed among the four canary seed cultivars. Interestingly, Snowbird wheat flour (0.65 mg FAE/g sample) contained about two-fold less TPC than canary seeds (1.34–1.47 mg/g sample), while oat had the highest content (2.04 mg FAE/g sample) of the cereals. Polyphenols are secondary metabolites produced by plants that play roles in defense mechanisms, primarily for protection against ultraviolet radiation [38]. These plant secondary metabolites are also useful as radical scavengers and possess positive biochemical effects against cardiovascular diseases, cancer growth, and age-related diseases [39]. Alfieri and Redaelli [40] recently analyzed twenty oat cultivars and reported their soluble phenol content (SPC) ranging from 0.78 to 1.09 mg GAE/g sample. Higher values, up to 1.5 mg GAE/g were found in oat grains by Adom and Liu [41], and a mean of 2.1 mg GAE/g was reported by Menga et al. [42]. Literature information on canary seeds is relatively scarce, despite the fact that a few studies that have investigated the phenolic profiles of hairy canary seed [32,43], as well as the phenolic profiles and antioxidant activities in germinated canary seed [44]. To the best of our knowledge, no data were previously published on these four hairless canary seeds cultivars to date.

### 3.2. Nutritional Quality of Canary Seed, Oat and Wheat Protein

#### 3.2.1. Amino Acid Composition 

The amino acid composition of the cereal flours is presented in Table 3. Between canary seed cultivars, there were only minor differences in amino acid composition. The yellow C09052 and C05041 cultivars had a significantly higher (*p* < 0.05) valine content (5.9 g/100 g) as compared to the brown cultivars (5.2 g/100 g). The isoleucine content was significantly higher (*p* < 0.05) in the brown cultivars (4.8 g/100 g) than the yellow C09052 cultivar (4.2 g/100 g), but not significantly different (*p* > 0.05) than the yellow C05041 variety (4.3 g/100 g). In general, the overall amino acid content was comparable to what has been previously reported by Abdel-Aal et al. [3] for hairy brown canary seeds and by the Canary Seed Development Comission of Saskatchewan [45] in hairless canary seed groats. However, both studies reported lower amounts of certain amino acids, such as lysine, isoleucine, valine, and histidine as compared to the current study, which is most probably due to breeding-induced improvement of the protein content and quality of these new experimental cultivars.

In general, the amino acid profile of canary seeds remains comparable to that of wheat and oat except for their tryptophan and valine content. Canary seeds cultivars had significantly higher (*p* < 0.05) amounts of valine (5.2–5.9 g/100 g) than both oat (3.4 g/100 g) and wheat (4.1 g/100 g) flours. Canary seed cultivars were also higher in arginine (5.7–6.1 g/100 g) compared to wheat (3.8 g/100 g). However, oat flour had significantly higher amounts of lysine (4.3 g/100 g) as compared to both canary seed (2.3–2.5 g/100 g protein) and wheat (2.2 g/100 g) flours. In cereal grains, the most limiting essential amino acid remains lysine. In comparison to other common cereals, canary seed has a similar lysine content to millet (2.8 g/100 g protein), but inferior to barley (3.9 g/100 g protein) [3,46]. Tryptophan, which is an essential amino acid normally lacking in cereals, makes canary seeds a valuable source with a content of 2.4–2.6 g/100 g, as compared to both oat (1.5 g/100 g) and wheat (1.1 g/100 g) flours. Therefore, combining canary seeds with other cereal grains would be an excellent practice to assure dietary demands for tryptophan in food formulations. In addition, canary seeds contain higher amounts of glutamic acid than oat, barley, and millet, as also reported by several other studies [3,46,47]. Glutamic acid is the most abundant amino acid in the brain, which plays significant roles in synaptic activity, memory, and learning, and its deficiency is related to the development of Alzheimer’s disease [48]. Moreover, high content of glutamic acid in the seeds could indicate the presence of high gamma-aminobutyric acid (GABA), a functional compound produced in plants primarily by the decarboxylation of L-glutamic acid, which has several health-promoting properties, including reducing blood pressure and blood cholesterol, anticancer, and anti-obesity activity [49]. GABA concentration, however, has not been directly determined in canary seeds. 

Finally as shown in Table 3, the total amino acid content of canary seeds (86.3–89.5 g/100 g) was not significantly different (*p* > 0.05) as compared to oat and wheat flours (86.2 g/100 g); however, the total essential amino acids (EAA) in canary seeds ranged between 34.2–35.7 g/100 g, which was significantly higher (*p* < 0.05) than the total essential amino acid content in oat (30.1 g/100 g) and wheat (29.3 g/100 g) flours, confirming that canary seeds would make an excellent addition to other cereal grain and legume products to ensure consumers meet the recommended dietary intake of essential amino acids.

#### 3.2.2. In Vitro Protein and Amino Acid Digestibility 

**Figure 1:**
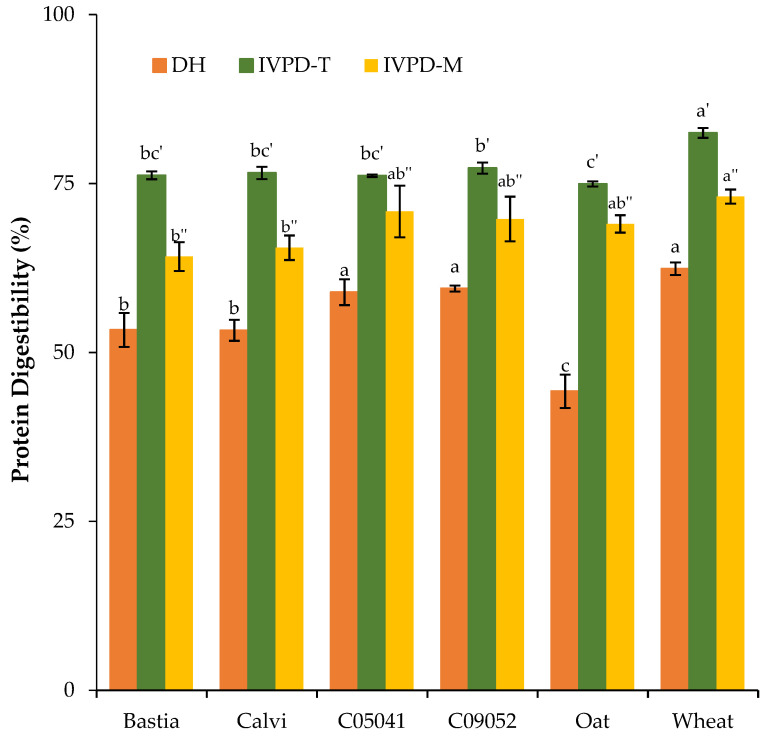
Protein digestibility of canary seed flours as compared to oat and wheat. Means with different lowercase letters within the same measured protein digestibility parameter (′, IVPD-T; ″, IVPD-M) are significantly different at *p* < 0.05 (n = 3); DH, Degree of hydrolysis; IVPD-T, in vitro protein digestibility as determined by pH-drop protocol [16]; IVPD-M, in vitro protein digestibility as determined by the INFOGEST protocol [17] and corresponding to the percent of the sum of total AAs recovered in the digestate supernatant compared to the total AAs in the cereal flour.

Protein digestibility is an important aspect of protein quality, since it provides information regarding the capacity of a protein to deliver amino acids to tissues and organs in the body [50]. Studied cereal flours were digested using two in vitro digestion protocols. The simplified pH-drop protocol of Tinus et al. [16] was used to determine the in vitro total protein digestibility (IVPD-T), and the complex harmonized in vitro digestion protocol INFOGEST of Minekus et al. [17] was used to assess ileal total protein digestibility (IVPD-M), which corresponded to the % of the sum of total AAs recovered in the digestate supernatant compared to the total AAs in the cereal flour. The in vitro true ileal digestibility for each AA (IV-TID), as well as the protein degree of hydrolysis (DH), were also determined following the INFOGEST protocol [27,28]. The DH was assessed as an indicator of the extent to which cereal proteins were hydrolyzed or digested. The results presented in Figure 1 show that the DH was significantly different (*p* < 0.001) among canary seed cultivars. The yellow cultivars (C05041 and C09052) had a mean DH of 59% as compared to 53% for brown cultivars (Bastia and Calvi). The brown canary seed cultivars had slightly higher trypsin inhibitor activity (TIA), which may explain the lower DH as compared to their counterparts. Wheat flour showed similar DH (64.2%) than C05041 and C09052 but significantly (*p* < 0.0001) higher DH than Bastia and Calvi. The DH of oat (44.3%) was significantly lower than the other studied cereals (*p* > 0.05). Similar DH values have been reported for sorghum (50.9–52.1%) [51] and buckwheat protein isolate (50.1–64.6%) [52]. 

The IVPD-T of canary seed flours was not significantly different among the studied varieties (*p* > 0.05) and ranged from 76.2% to 77.3% (Figure 1). Wheat had the highest IVPD-T value (82.5%), whereas oat had the lowest (75.0%) one. Similar IVPD values were observed for pea protein concentrate, lentil, pinto bean, and buckwheat flours as compared to canary seed flours [53,54,55] using the same pH-drop method of Tinus et al. [16]. In addition, for all cereal varieties, the IVPD values determined by the pH-drop method (IVPD-T) were higher than those obtained by the INFOGEST protocol (IVPD-M). The two digestion protocols use different enzymes, enzyme to substrate ratios, hydrolysis times, and reaction conditions, which may explain the difference in IVPD values between the two methods. Nevertheless, a regression analysis showed that IVPD-T and IVPD-M values have a positive and significant correlation as indicated by a Pearson correlation coefficient (r^2^) of 0.5 and *p*-values of 0.035. The pH-drop in vitro digestion protocol is a simple model to simulate the digestion process, where a mixture solution of trypsin, chymotrypsin and protease is added to the same test tube, versus the more complex INFOGEST model which comprises three digestion phases (oral, gastric, and intestinal). The conditions used during each digestion phase (pH, duration, digestive fluid composition, and enzymatic activities) are a scientific consensus based on up-to-date physiological data. Due to its low cost and relatively simple execution, the pH-drop digestion model could be suitable for preliminary screening or comparative studies or where a high number of samples are to be analyzed. The INFOGEST model, instead, reflects better the complexity of the digestion process that occurs in vivo, and makes it possible to determine, in more physiologically representative conditions, the digestibility for each amino acid allowing for the calculation of protein in vitro digestible indispensable amino acid score (DIAAS). The IVPD-M and the DH were also positively correlated with a r^2^ and *p*-value of 0.67 and 0.002, respectively. As expected IVPD-M negatively correlated with trypsin inhibitor content of the cereal flour (r^2^ = −0.52 and *p*-value = 0.035) but did not show significant correlation with phytate content. 

#### 3.2.3. Calculation of PDCAAS and DIAAS Scores 

The digestible indispensable amino acid score (DIAAS) and the protein digestibility corrected amino acid score (PDCAAS) values are measurements of protein quality and provide information regrading the ability of a specific protein to provide sufficient amounts of essential amino acids for human requirements [7]. In the present study, and to allow for comparison, both PDCAAS and DIAAS scores were calculated based on the ratio of the amount of the first-limiting dietary indispensable amino acid in the protein source to the amino acid requirement for infants, children, older children/adolescents/adults (Table 1) as suggested by the FAO expert committee report in 2013 [8]. The in vitro PDACAAS was corrected for protein digestibility determined either by the Tinus et al. [16] method (PDCAAS-T) or the Minekus et al. [17] method (PDCAAS-M) (Table 4). 

As expected, the results indicate that for all studied cereals, lysine was the limiting essential amino acid (Table 4). For the PDCAAS values, no significant difference (*p* > 0.05) was observed between the brown and yellow canary seed cultivars. Overall, canary seed varieties have similar PDCAAS values to wheat, even though PDCAAS-T values appear slightly higher than those of wheat but not statistically significant. Oat, however, exhibited significantly higher PDCAAS values among the studied cereal grains, which is in good agreement with its higher amount of lysine (Table 3) as compared to wheat and canary seeds. The determined oat PDCAAS-T and PDCAAS-M values of 57.1 (Lys) and 52.6 (Lys) for children (6 months–3 years) are similar to the PDCAAS value of 58 (Lys) reported by Mathai et al. [10] and Abelilla, et al. [56] for the same age group. Their determined PDCAAS value for wheat of 51 (Lys) is, however, higher than what was determined for wheat (32(Lys)) in this study. Additionally, our findings are in line with those of Cervantes-Pahm et al. [57], who reported that dehulled oats have a greater apparent ileal digestibility and total amino acids (AA) content than most other cereal grains in a pig model. Overall, the obtained PDCAAS values suggested the nutritional quality of canary seeds was slightly better than wheat due to its higher lysine AA score (lysine content), but lower than oat. 

For the calculation of the DIAAS, the minimum true ileal digestibility (IV-TID) values for each amino acid were determined following the INFOGEST protocol [17] by measuring the free amino acids present in the digestate, while maximum IV-TID values were calculated from the total amino acids released by acid hydrolysis of the whole digestate (comprising free amino acids, polypeptides, and soluble proteins). Since not all digested AA are in readily bioavailable form (free amino acids), it is taught that true ileal digestibility could be a value between the minimum and maximum IV-TID values. Overall, as shown in Table 5**,** both minimum and maximum amino acid IV-TID were comparable within the two brown and the two yellow canary seed cultivars. However, minimum IV-TID values for most of the AA, particularly for cysteine, tyrosine, and aromatic amino acids, were higher for the yellow cultivars than the brown. Among the canary seed cultivars, the IV-TID of most AA was higher (*p* < 0.05) in yellow C05041 variety. The total amount of released free amino acid was also higher for this variety (30%) in comparison to the brown Bastia variety (26%). This might be related to a difference in protein exposure to the digestive enzymes between the yellow and brown canary seed varieties due to some undefined interactions. As shown in Table 5, 26–30% of canary seed proteins were digested to the free amino acid level following the INFOGEST digestion of canary seed flours compared to 32.5% for oat and 21.6% for wheat. As expected, the total AA maximum TID values increased for all studied cereals ranging from 64–71% for canary cultivars and 67.5% for oat and 71.8% for wheat. As well, the maximum TID values for each amino acid were greater than the calculated minimum TID values. This increase is attributed to the added contribution of polypeptides and soluble proteins present in the digestate. Overall, no significant difference (*p* > 0.05) in the total AA TID was found between the studied cereals. The minimum and maximum TID for the essential amino acids, lysine, histidine, and threonine were, however, significantly lower (*p* < 0.05) in canary seeds compared to oat and wheat. Moreover, some discrepancies were noted for the aromatic amino acids in canary seeds and oat, where the minimum TID values were inferior to the maximum TID values. This could be explained by a possible degradation and loss of some of these AAs during the acidic hydrolysis leading to underestimated values. Individual proteins, depending on their amino acid sequence, will produce different rates of amino acid liberation and loss during acidic protein hydrolysis [58,59]. Minimum TID values for amino acids, such as lysine, methionine, and threonine for the canary seed varieties and lysine for wheat were not significantly different (*p* > 0.05) from their corresponding maximum TID values, suggesting the digestibility of these particular amino acids are low for these cereal grains. In addition, the maximum TID values reported for cysteine are overestimated (137–298%) for canary seed, oat, and wheat samples, which could be due to the used AA analytical method being not suitable for the determination of sulfur amino acids in protein hydrolysates. The data on cysteine digestibility, however, would have no impact on DIAAS calculations in this particular study, as they are not the limiting AA in cereals.

The data obtained for minimum and maximum IV-TID were used to calculate their corresponding minimum and maximum DIAAS based on the three scoring patterns recommended by the FAO [8]. As shown in Table 6 and Table 7, the minimum DIAAS scores in canary seeds for infants, children, and older children/adolescents/adults ranged from 5–8, 6–9, and 8–11, respectively. The maximum DIAAS scores in canary seeds for infants, children, and older children/adolescents/adults ranged from 8–10, 9–12, and 11–14, respectively. Between canary seed cultivars, there was no significant difference (*p* > 0.05) in DIAAS values. Overall, canary seed DIAAS scores (Table 6 and Table 7) appear to be lower than those of wheat (12–15, 14–18, and 17–22 for infants, children, and adults, respectively) and oat (20–34, 25–41, and 29–49, for infants, children, and adults, respectively), which exhibited significantly higher (*p* > 0.05) DIAAS values. 

The protein quality of canary seed flours as indicated by the DIAAS scores are low, with lysine being the limiting amino acid. DIAAS values have been calculated for many cereal grains, but it remains difficult to compare these values since different methods were used to determine the TID of amino acids. Cervantes-Pahm et al. [57] reported DIAAS values (determined from growing pigs) for older children, adolescents, and adults for barley (51), oat (77), rye (47), sorghum (29), and wheat (43), for which lysine was the limiting amino acid in each grain. These values are significantly higher than the IV-DIAAS values determined for oat and wheat in the present study. Han et al. [60], however, reported DIAAS values (determined from growing rats) for older children, adolescents, and adults for cooked brown rice (42), buckwheat (68), oats (43), millet (10), adlay (13), and whole wheat (20), for which lysine was also the limiting amino acid in each grain except for buckwheat (SAA). In the present study, the in vitro DIAAS values determined for canary seeds in children (8–12) are similar to those reported for millet and adlay, which as canary seeds, all belong to the grass family *Poaceae*. For all the above-mentioned studies (including the present study), oats had a higher DIAAS value than wheat and one of the highest among common cereal grains. The presence of higher content of phytate in canary seed compared to wheat and oat (Table 2) may contribute to lowering canary seed digestibility and DIAAS values as phytate was shown to form complexes with proteins, proteases, and amylases, inhibiting proteolysis [35,61].

## 4. Conclusions

**Figure 2:**
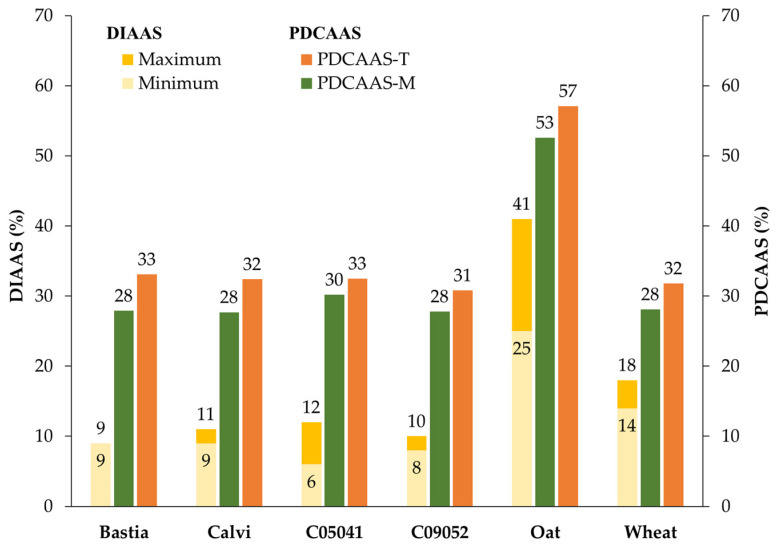
Protein nutritional quality scores for canary seed, wheat, and oat based on in vitro digestion models. DIAAS maximum and minimum and digestible indispensable amino acid score determined based on true ileal digestibility of total amino acid and free amino acid, respectively, by the INFOGEST protocol [17]; PDCAAS-M: protein digestibility corrected amino acid score obtained with the INFOGEST protocol [17]; PDCAAS-T: protein digestibility corrected amino acid score obtained with the pH-drop method [16].

The protein content of canary seeds is high as compared to other cereals, including oat and wheat. The nutritional quality of a protein depends not only on protein quantity but the ability to cover human dietary requirements for bioavailable and digestible essential amino acids. As summarized in Figure 2, the in vitro PDCAAS and DIAAS scores for canary seeds were lower than oat, but comparable to those of wheat. The low protein quality scores of canary seed are attributed to their lower lysine content as well as to lower lysine digestibility in the seeds. Similarly to other cereals, canary seeds must be consumed with other proteins sources in order to complete nutritional requirements for essential amino acids. They have, however, higher amounts of tryptophan than other cereals, and could be combined with these to improve their overall quality.

The in vitro digestion models used in this study, despite some limitations, have proven their utility as rapid and simple approaches to compare protein quality scores between different sources. Future studies should evaluate the addition of enzymes from the brush border membranes, including peptidases to the INFOGEST model, to better simulate human physiological conditions and enhance amino acid digestibility. Further standardization, validation, and correlation studies with in vivo data are still needed to advance the adoption of these models as standardized and robust approaches for the evaluation of protein quality especially for screening purposes in the development and assessment of novel proteins and food formulations, and which is highly recommended by the FAO/WHO [8].

## Figures and Tables

**Table 1:** Recommended amino acid reference pattern for infants, children, older children/adolescents/adults [8].

	Age Group		
Essential amino acid	Infant (0–6 months)	Child (6 months–3 years)	Older child/ adolescent/adult
	Reference Pattern (mg/g protein)		
His	21	20	16
Ile	55	32	30
Leu	96	66	61
Lys	69	57	48
SAA (Cys + Met)	33	27	23
AAA (Phe + Tyr)	94	52	41
Thr	44	31	25
Trp	17	8.5	6.6
Val	55	43	40

**Table 2:** Protein content and anti-nutritional components of hairless canary seed, oat, and wheat flours.

Cereal Variety	Brown Canary Seeds		Yellow Canary Seeds		Oat	Wheat
	Bastia	Calvi	C05041	C09052		
Protein (%)	22.33 ± 0.26 ^a^	21.55 ± 0.10 ^a^	21.53 ± 0.25 ^a^	21.97 ± 0.56 ^a^	14.30 ± 0.12 ^c^	16.35 ± 0.07 ^b^
TIA (mg/g)	0.15 ± 0.001 ^ab^	0.16 ± 0.005 ^a^	0.12 ± 0.005 ^ab^	0.13 ± 0.003 ^ab^	0.14 ± 0.01 ^ab^	0.11 ± 0.035 ^b^
Phytate (mg/g)	12.36 ± 0.63 ^a^	12.12 ± 1.32 ^a^	11.96 ± 0.42 ^a^	12.17 ± 0.45 ^a^	5.94 ± 0.20 ^b^	3.14 ± 0.07 ^c^
TPC (mg FAE/g)	1.44 ± 0.03 ^b^	1.42 ± 0.01 ^b^	1.34 ± 0.06 ^b^	1.47 ± 0.01 ^b^	2.04 ± 0.15 ^a^	0.65 ± 0.05 ^c^

Means in a row with different lowercase letters are significantly different at *p* < 0.05 (*n* = 3). TIA: Trypsin inhibitor activity expressed as mg of pure trypsin inhibited per gram of sample. Phytate: mg of phytate per gram of sample. TPC: Total polyphenol content expressed as mg of ferulic acid equivalents per g of sample.

**Table 3:** Amino acid composition of hairless canary seed, oat, and wheat flours (g/100 g protein).

Amino Acid	Cereal Variety					
	Brown Canary Seeds		Yellow Canary Seeds		Oat	Wheat
	Bastia	Calvi	C05041	C09052		
Asp	4.47 ± 0.07 ^b^	4.47 ± 0.02 ^b^	4.52 ± 0.18 ^b^	4.39 ± 0.25 ^b^	9.04 ± 0.16 ^a^	4.09 ± 0.3 ^b^
Glu	26.56 ± 0.80 ^c^	28.63 ± 0.96 ^bc^	28.60 ± 0.2 ^bc^	28.84 ± 0.79 ^b^	22.18 ± 0.37 ^d^	35.04 ± 1.15 ^a^
Ser	3.28 ± 0.13 ^b^	4.45 ± 0.12 ^b^	3.25 ± 0.0 ^b^	3.40 ± 0.12 ^b^	5.01 ± 0.06 ^a^	4.64 ± 0.31 ^a^
His *	2.64 ± 0.24 ^a^	2.69 ± 0.22 ^a^	2.65 ± 0.23 ^a^	2.63 ± 0.23 ^a^	2.41 ± 0.03 ^a^	2.56 ± 0.12 ^a^
Gly	2.98 ± 0.19 ^b^	2.82 ± 0.23 ^bc^	2.71 ± 0.18 ^bc^	2.52 ± 0.03 ^c^	4.28 ± 3.27 ^a^	2.73 ± 0.17 ^bc^
Thr *	2.95 ± 0.15 ^ab^	2.88 ± 0.18 ^ab^	2.99 ± 0.04 ^ab^	2.55 ± 0.27 ^bc^	3.27 ± 0.04 ^a^	2.33 ± 0.24 ^c^
Arg	6.02 ± 0.13 ^a^	6.13 ± 0.17 ^a^	5.78 ± 0.09 ^a^	5.66 ± 0.31 ^a^	6.17 ± 0.10 ^a^	3.81 ± 0.41 ^b^
Ala	4.10 ± 0.13 ^a^	4.28 ± 0.21 ^a^	4.07 ± 0.07 ^a^	4.09 ± 0.10 ^a^	4.45 ± 0.04 ^a^	2.88 ± 0.34 ^b^
Tyr	2.37 ± 0.11 ^b^	2.48 ± 0.18 ^b^	2.18 ± 0.17 ^b^	2.12 ± 0.19 ^b^	3.41 ± 0.06 ^a^	3.14 ± 0.17 ^a^
Cys	1.36± 0.29 ^abc^	1.59 ± 0.20 ^a^	1.15 ± 0.03 ^bc^	1.04 ± 0.11 ^c^	1.51 ± 0.04 ^ab^	0.56 ± 0.00 ^d^
Val *	5.19 ± 0.20 ^b^	5.15 ± 0.02 ^b^	5.91 ± 0.18 ^a^	5.93 ± 0.06 ^a^	3.43 ± 0.07 ^d^	4.08 ± 0.26 ^c^
Met *	0.97 ± 0.12 ^a^	1.04 ± 0.10 ^a^	1.06 ± 0.04 ^a^	0.95 ± 0.06 ^a^	0.89 ± 0.22 ^a^	0.97 ± 0.02 ^a^
Trp *	2.46 ± 0.07 ^bc^	2.56 ± 0.04 ^ab^	2.44 ± 0.01 ^c^	2.60 ± 0.02 ^a^	1.50 ± 0.05 ^d^	1.11 ± 0.04 ^e^
Phe *	6.19 ± 0.17 ^a^	6.23 ± 0.17 ^a^	6.02 ± 0.12 ^a^	5.68 ± 0.04 ^ab^	5.11 ± 0.08 ^b^	5.27 ± 0.24 ^b^
Ile *	4.83 ± 0.30 ^a^	4.85 ± 0.17 ^a^	4.31 ± 0.13 ^ab^	4.16 ± 0.28 ^b^	2.49 ± 0.01 ^c^	3.80 ± 0.20 ^b^
Leu *	7.69 ± 0.22 ^ab^	7.86 ± 0.37 ^a^	7.37 ± 0.08 ^ab^	7.43 ± 0.21 ^ab^	6.66 ± 0.09 ^c^	7.02 ± 0.37 ^bc^
Lys *	2.48 ± 0.12 ^b^	2.41 ± 0.06 ^b^	2.43 ± 0.19 ^b^	2.27 ± 0.04 ^b^	4.34 ± 0.24 ^a^	2.19 ± 0.11 ^b^
Total AA	86.54 ± 0.85 ^a^	89.53 ± 2.65 ^a^	87.44 ± 0.84 ^a^	86.27 ± 1.91 ^a^	86.15 ± 0.60 ^a^	86.24 ± 2.18 ^a^
Total EAA	35.40 ± 0.99 ^a^	35.67 ± 1.13 ^a^	35.18 ± 0.32 ^a^	34.22 ± 1.35 ^a^	30.10 ± 0.15 ^b^	29.34 ± 0.71 ^b^

Means in a row with different lowercase letters are significantly different at *p* < 0.05 (*n* = 3); * Essential amino acid.

**Table 4:** In vitro protein digestibility corrected amino acid score (PDCAAS) of cereal flours.

	Canary Seeds				Oat	Wheat	*p*-Value
	Bastia	Calvi	C05041	C09052			
Infant (0–6 months)							
AA score	0.36 ^b^	0.35 ^b^	0.35 ^b^	0.33 ^b^	0.63 ^a^	0.32 ^b^	<0.0001
Limiting AA	Lysine	Lysine	Lysine	Lysine	Lysine	Lysine	
PDCAAS-T	27.3 ^b^	26.8 ^b^	26.8 ^b^	25.4 ^b^	47.2 ^a^	26.2 ^b^	<0.0001
PDCAAS-M	23.0 ^b^	22.9 ^b^	25.0 ^b^	22.9 ^b^	43.5 ^a^	23.2 ^b^	<0.0001
Child (6 months–3 years)							
AA score	0.43 ^b^	0.42 ^b^	0.43 ^b^	0.40 ^b^	0.76 ^a^	0.39 ^b^	<0.0001
Limiting AA	Lysine	Lysine	Lysine	Lysine	Lysine	Lysine	
PDCAAS-T	33.1 ^b^	32.4 ^b^	32.5 ^b^	30.8 ^b^	57.1 ^a^	31.8 ^b^	<0.0001
PDCAAS-M	27.9 ^b^	27.7 ^b^	30.2 ^b^	27.8 ^b^	52.6 ^a^	28.1 ^b^	<0.0001
Older children, adolescents, adults							
AA score	0.52 ^b^	0.50 ^b^	0.51 ^b^	0.47 ^b^	0.91 ^a^	0.46 ^b^	<0.0001
Limiting AA	Lysine	Lysine	Lysine	Lysine	Lysine	Lysine	
PDCAAS-T	39.3 ^b^	38.5 ^b^	38.6 ^b^	36.6 ^b^	67.8 ^a^	37.7 ^b^	<0.0001
PDCAAS-M	33.1 ^b^	32.9 ^b^	35.9 ^b^	33.0 ^b^	62.5 ^b^	33.4 ^b^	<0.0001

Means in a row with different lowercase letters are significantly different at *p* < 0.05 (n = 3); AA score: content of the first limiting amino acid in the test protein (mg/g protein)/corresponding content of this amino acid according to FAO reference pattern (mg/g protein) as reported in Table 1. PDCAAS-T: product of AA score and IVPD-T (%) obtained with the pH-drop method of Tinus, et al. [16]. PDCAAS-M: product of AA score and IVPD-M (%) obtained with the INFOGEST method of Minekus, et al. [17].

**Table 5:** Minimum and maximum in vitro amino acid true ileal digestibility (IV-TID) (%) of hairless canary seed, oat, and wheat flours.

**Minimum IV-TID (%) = Free AAs in Digestate Supernatant**							
Amino Acid	Cereal Variety						
	Bastia	Calvi	C05041	C09052	Oat	Wheat	*p*-value
Ala	27.15 ^b^	28.78 ^b^	40.20 ^a^	32.45 ^ab^	29.70 ^b^	28.53 ^b^	0.0004
Arg	71.59 ^c^	85.22 ^b^	90.79 ^b^	85.00 ^b^	105.20 ^a^	116.00 ^a^	0.0000
Asp	0.90 ^b^	0.83 ^b^	1.19 ^b^	0.40 ^b^	6.29 ^a^	7.19 ^a^	0.0000
Cys	2.96 ^c^	2.61 ^c^	7.74 ^c^	7.97 ^c^	19.64 ^b^	51.13 ^a^	0.0000
Glu	0.04 ^b^	0.15 ^b^	0.52 ^b^	0.17 ^b^	3.70 ^b^	0.92 ^ab^	0.0000
Gly	5.24 ^b^	5.97 ^b^	9.39 ^ab^	9.08 ^ab^	5.66 ^b^	11.84 ^a^	0.0014
His	5.95 ^b^	5.14 ^b^	5.53 ^b^	5.57 ^b^	27.81 ^a^	19.04 ^a^	0.0004
Ile	38.78 ^c^	38.19 ^c^	44.55 ^b^	39.36 ^bc^	61.43 ^a^	38.14 ^c^	0.0000
Leu	55.75 ^b^	59.10 ^ab^	62.40 ^a^	57.26 ^b^	49.43 ^c^	36.83 ^d^	0.0000
Lys	23.21 ^b^	18.06 ^bc^	12.75 ^c^	17.35 ^bc^	32.28 ^a^	36.55 ^ab^	0.0002
Met	87.08 ^bc^	105.08 ^a^	73.31 ^cd^	90.24 ^b^	93.26 ^ab^	59.58 ^d^	0.0000
Phe	59.23 ^ab^	58.69 ^b^	66.36 ^a^	60.43 ^ab^	57.51 ^b^	35.05 ^c^	0.0000
Ser	9.11 ^b^	10.28 ^b^	16.05 ^a^	13.38 ^ab^	12.66 ^ab^	12.17 ^ab^	0.0038
Thr	17.99 ^de^	17.65 ^e^	27.51 ^bc^	22.75 ^cd^	45.22 ^ab^	41.55 ^a^	0.0000
Tyr	75.98 ^c^	70.04 ^c^	104.09 ^ab^	106.57 ^a^	93.31 ^b^	44.00 ^d^	0.0000
Val	37.19 ^b^	34.87 ^bc^	28.93 ^de^	27.22 ^e^	50.91 ^a^	31.36 ^cd^	0.0000
SAA (Cys + Met)	53.39 ^a^	57.36 ^a^	52.83 ^a^	57.63 ^a^	47.03 ^a^	56.49 ^a^	0.0560
AAA (Phe + Tyr)	63.86 ^b^	61.87 ^b^	76.93 ^a^	72.76 ^a^	71.83 ^a^	38.39 ^c^	0.0000
∑ Free AAs digestibility	25.93 ^c^	26.45 ^bc^	29.78 ^ab^	27.96 ^bc^	32.42 ^a^	21.63 ^d^	0.0000
**Maximum IV-TID (%) = Total AAs in digestate supernatant**							
Amino Acid	Cereal Variety						
	Bastia	Calvi	C05041	C09052	Oat	Wheat	
Ala	73.28 ^d^	73.26 ^d^	88.93 ^b^	82.83 ^bc^	76.35 ^cd^	100.60 ^a^	0.0000
Arg	90.02 ^ab^	98.20 ^a^	100.94 ^a^	100.44 ^a^	66.27 ^c^	84.78 ^b^	0.0000
Asp	40.79 ^c^	48.00 ^bc^	49.13 ^bc^	44.50 ^bc^	58.77 ^b^	88.56 ^a^	0.0000
Cys	210.41 ^bc^	198.19 ^c^	249.11 ^b^	218.71 ^bc^	137.03 ^d^	298.13 ^a^	0.0000
Glu	61.14 ^b^	62.57 ^b^	65.42 ^ab^	67.17 ^ab^	66.19 ^ab^	68.80 ^a^	0.0113
Gly	96.13 ^bc^	108.31 ^ab^	131.97 ^ab^	146.12 ^a^	27.12 ^d^	56.41 ^cd^	0.0000
His	22.34 ^c^	19.88 ^c^	30.08 ^b^	25.69 ^bc^	90.57 ^a^	77.38 ^a^	0.0000
Ile	47.59 ^d^	47.70 ^d^	57.68 ^c^	52.17 ^d^	114.46 ^a^	72.41 ^b^	0.0000
Leu	88.50 ^b^	101.46 ^a^	102.65 ^a^	96.67 ^ab^	63.22 ^c^	60.14 ^c^	0.0000
Lys	23.99 ^b^	20.80 ^b^	23.21 ^b^	20.34 ^b^	53.60 ^a^	47.13 ^a^	0.0000
Met	87.64 ^b^	81.17 ^b^	90.97 ^b^	84.57 ^b^	149.89 ^a^	135.50 ^a^	0.0000
Phe	65.14 ^cd^	62.98 ^d^	76.59 ^b^	70.51 ^bc^	85.30 ^a^	77.29 ^b^	0.0000
Ser	102.24 ^ab^	92.08 ^b^	103.70 ^a^	111.32 ^a^	61.13 ^c^	65.78 ^c^	0.0000
Thr	16.60 ^c^	17.64 ^c^	21.70 ^c^	20.08 ^c^	77.01 ^b^	98.40 ^a^	0.0000
Tyr	24.08 ^d^	25.37 ^cd^	43.29 ^ab^	35.69 ^bc^	42.35 ^ab^	39.49 ^a^	0.0001
Val	50.42 ^c^	48.93 ^c^	40.20 ^d^	39.77 ^d^	100.09 ^a^	77.96 ^b^	0.0000
SAA (Cys + Met)	136.80 ^b^	135.67 ^b^	140.36 ^b^	137.74 ^b^	141.81 ^b^	195.11 ^a^	0.0000
AAA (Phe + Tyr)	53.80 ^bc^	52.45 ^b^	67.26 ^a^	61.21 ^ab^	53.24 ^c^	50.00 ^c^	0.0000
∑ Free AAs digestibility	64.19 ^a^	65.54 ^a^	70.87 ^a^	69.75 ^a^	67.53 ^a^	71.78 ^a^	0.0343

Means in a row with different lowercase letters are significantly different at *p* < 0.05 (*n* = 3). SAA: sulfur amino acid; AAA: aromatic amino acid. Minimum TID was calculated as the free amino acids (AAs) content in mg per g protein in supernatant digestate/sum of total AAs (mg)/g protein in cereal flour; while maximum TID was calculated as sum of total AAs (mg)/per g protein in supernatant digestate/sum of total AAs (mg)/g protein in cereal flour.

**Table 6:** Minimum digestible indispensable amino acid score (DIAAS) of canary seed, oat, and wheat proteins determined from free (bioaccessible) amino acids after in vitro digestion.

	Indispensable Amino Acids (IAA)								
Cereal variety	His	Thr	Val	Ile	Leu	Lys	SAA	AAA	DIAAS, %
	Infant DIAA reference ratio (0–6 months)								
Bastia	0.08 ^b^	0.11 ^b^	0.34 ^ab^	0.33 ^a^	0.49 ^a^	0.08 ^b^	0.29 ^ab^	0.66 ^b^	8 (Lys) ^b^
Calvi	0.08 ^b^	0.12 ^b^	0.37 ^a^	0.36 ^a^	0.50 ^a^	0.08 ^bc^	0.35 ^a^	0.71 ^ab^	8 (Lys) ^bc^
C05041	0.06 ^b^	0.13 ^b^	0.37 ^a^	0.35 ^a^	0.50 ^a^	0.05 ^c^	0.31 ^ab^	0.73 ^ab^	5 (Lys) ^c^
C09052	0.07 ^b^	0.12 ^b^	0.35 ^ab^	0.33 ^a^	0.49 ^a^	0.07 ^bc^	0.32 ^ab^	0.72 ^a^	7 (Lys) ^bc^
Oat	0.32 ^a^	0.34 ^a^	0.32 ^b^	0.28 ^b^	0.34 ^b^	0.20 ^a^	0.34 ^a^	0.64 ^b^	20 (Lys) ^a^
Wheat	0.23 ^a^	0.22 ^a^	0.23 ^c^	0.26 ^b^	0.27 ^c^	0.12 ^b^	0.26 ^b^	0.34 ^c^	12 (Lys) ^b^
*p*-value	0.0006	0.0001	0	0	0	0.0001	0.0077	0	0.0001
	Child DIAA reference ratio (6 months–3 years)								
Bastia	0.09 ^b^	0.15 ^b^	0.43 ^ab^	0.57 ^a^	0.71 ^a^	0.09 ^b^	0.35 ^ab^	1.20 ^a^	9 (Lys) ^b^
Calvi	0.09 ^b^	0.17 ^b^	0.48 ^a^	0.62 ^a^	0.73 ^a^	0.09 ^bc^	0.42 ^a^	1.29 ^a^	9 (Lys) ^bc^
C05041	0.07 ^b^	0.18 ^b^	0.47 ^a^	0.60 ^a^	0.73 ^a^	0.06 ^c^	0.38 ^ab^	1.33 ^a^	6 (Lys) ^c^
C09052	0.08 ^b^	0.17 ^b^	0.44 ^ab^	0.56 ^a^	0.71 ^a^	0.08 ^bc^	0.40 ^ab^	1.31 ^a^	8 (Lys) ^bc^
Oat	0.33 ^a^	0.48 ^a^	0.41 ^b^	0.48 ^b^	0.50 ^b^	0.25 ^a^	0.41 ^a^	1.16 ^a^	25 (Lys) ^a^
Wheat	0.24 ^a^	0.31 ^a^	0.30 ^c^	0.45 ^b^	0.39 ^c^	0.14 ^b^	0.32 ^b^	0.62 ^b^	14 (Lys) ^b^
*p*-value	0	0	0	0	0	0	0.0077	0	0
	Older child, adolescent, adult DIAA reference ratio								
Bastia	0.11 ^b^	0.19 ^b^	0.47 ^ab^	0.61 ^a^	0.77 ^a^	0.11 ^b^	0.41 ^ab^	1.52 ^a^	11 (Lys) ^b^
Calvi	0.09 ^b^	0.23 ^b^	0.51 ^a^	0.64 ^a^	0.79 ^a^	0.08 ^bc^	0.45 ^a^	1.68 ^a^	11 (Lys) ^bc^
C05041	0.11 ^b^	0.21 ^b^	0.51 ^a^	0.67 ^a^	0.79 ^a^	0.11 ^c^	0.50 ^ab^	1.63 ^a^	8 (Lys) ^c^
C09052	0.10 ^b^	0.21 ^b^	0.48 ^ab^	0.60 ^a^	0.77 ^a^	0.10 ^bc^	0.46 ^ab^	1.66 ^a^	10 (Lys) ^bc^
Oat	0.42 ^a^	0.59 ^a^	0.44 ^b^	0.51 ^b^	0.54 ^b^	0.29 ^a^	0.48 ^a^	1.48 ^a^	29 (Lys) ^a^
Wheat	0.30 ^a^	0.39 ^a^	0.32 ^c^	0.48 ^b^	0.42 ^c^	0.17 ^b^	0.37 ^b^	0.79 ^b^	17 (Lys) ^b^
*p*-value	0	0	0	0	0	0	0.0077	0	0

Means in a row with different lowercase letters are significantly different at *p* < 0.05 (n = 3). IAA: indispensable amino acid. DIAA reference ratio: ratio of the digestible indispensable amino acid content (mg/g protein) in the test protein to the corresponding content of the same amino acid in the FAO reference pattern (mg/g protein) (Table 1).

**Table 7:** Maximum digestible indispensable amino acid score (DIAAS) of canary seed, oat, and wheat proteins determined from free amino acids after in vitro digestion.

	Indispensable Amino Acids (IAA)								
Cereal variety	His	Thr	Val	Ile	Leu	Lys	SAA	AAA	DIAAS, %
	Infant DIAA reference ratio (0–6 months)								
Bastia	0.31 ^b^	0.10 ^b^	0.46 ^c^	0.41 ^b^	0.77 ^b^	0.08 ^c^	0.73 ^c^	0.56 ^ab^	8 (Lys ) ^c^
Calvi	0.32 ^b^	0.12 ^b^	0.52 ^bc^	0.45 ^b^	0.86 ^a^	0.09 ^bc^	0.82 ^bc^	0.60 ^ab^	9 (Lys) ^bc^
C05041	0.35 ^b^	0.10 ^b^	0.51 ^bc^	0.45 ^b^	0.83 ^ab^	0.10 ^bc^	0.83 ^bc^	0.64 ^a^	10 (Lys) ^bc^
C09052	0.34 ^b^	0.10 ^b^	0.50 ^bc^	0.43 ^b^	0.82 ^ab^	0.08 ^c^	0.77 ^bc^	0.61 ^a^	8 (Lys) ^c^
Oat	1.04 ^a^	0.57 ^a^	0.63 ^a^	0.52 ^a^	0.44 ^c^	0.34 ^a^	1.03 ^a^	0.48 ^bc^	34 (Lys) ^a^
Wheat	0.94 ^a^	0.52 ^a^	0.58 ^ab^	0.50 ^a^	0.44 ^c^	0.15 ^b^	0.90 ^ab^	0.45 ^c^	15 (Lys) ^b^
*p*-value	0	0	0.0004	0	0	0	0.0004	0	0
	Child DIAA reference ratio (6 months–3 years)								
Bastia	0.31 ^b^	0.14 ^b^	0.57 ^c^	0.68 ^c^	1.10 ^b^	0.09 ^c^	0.86 ^c^	0.93 ^abc^	9 (Lys) ^c^
Calvi	0.33 ^b^	0.17 ^b^	0.67 ^bc^	0.78 ^b^	1.25 ^a^	0.11 ^bc^	1.00 ^bc^	1.09 ^ab^	11 (Lys) ^bc^
C05041	0.37 ^b^	0.14 ^b^	0.66 ^bc^	0.77 ^b^	1.20 ^ab^	0.12 ^bc^	1.02 ^bc^	1.16 ^a^	12 (Lys) ^bc^
C09052	0.36 ^b^	0.15 ^b^	0.65 ^bc^	0.74 ^bc^	1.20 ^ab^	0.10 ^c^	0.94 ^bc^	1.10 ^b^	10 (Lys) ^c^
Oat	1.09 ^a^	0.81 ^a^	0.80 ^a^	0.89 ^a^	0.64 ^c^	0.41 ^a^	1.26 ^a^	0.87 ^bc^	41 (Lys) ^a^
Wheat	0.99 ^a^	0.74 ^a^	0.74 ^ab^	0.86 ^a^	0.64 ^c^	0.18 ^b^	1.10 ^ab^	0.81 ^c^	18 (Lys) ^b^
*p*-value	0	0	0.0001	0	0	0	0.0001	0	0
	Older child, adolescent, adult DIAA reference ratio								
Bastia	0.39 ^b^	0.17 ^b^	0.61 ^c^	0.72 ^c^	1.19 ^b^	0.11 ^c^	1.01 ^c^	1.18 ^abc^	11 (Lys) ^c^
Calvi	0.42 ^b^	0.21 ^b^	0.72 ^bc^	0.83 ^b^	1.36 ^a^	0.13 ^bc^	1.17 ^bc^	1.39 ^ab^	13 (Lys) ^bc^
C05041	0.46 ^b^	0.18 ^b^	0.70 ^bc^	0.83 ^b^	1.30 ^ab^	0.14 ^bc^	1.20 ^bc^	1.47 ^a^	14 (Lys) ^bc^
C09052	0.45 ^b^	0.18 ^b^	0.69 ^bc^	0.79 ^bc^	1.30 ^ab^	0.12 ^c^	1.11 ^bc^	1.39 ^a^	12 (Lys) ^c^
Oat	1.36 ^a^	1.01 ^a^	0.86 ^a^	0.95 ^a^	0.69 ^c^	0.49 ^a^	1.48 ^a^	1.11 ^bc^	49 (Lys) ^a^
Wheat	1.24 ^a^	0.92 ^a^	0.80 ^ab^	0.92 ^a^	0.69 ^c^	0.22 ^b^	1.29 ^ab^	1.03 ^c^	22 (Lys) ^b^
*p*-value	0	0	0.0001	0	0	0	0.0001	0	0

Means in a row with different lowercase letters are significantly different at *p* < 0.05 (*n* = 3). IAA: indispensable amino acid. DIAA reference ratio: ratio of the digestible indispensable amino acid content (mg/g protein) in the test protein to the corresponding content of the same amino acid in the FAO reference pattern (mg/g protein [8]) (Table 1).

## Data Availability

The data presented in this study are available on request from the corresponding author.
